# The complete mitochondrial genome of *Syrista parreyssii* (Spinola, 1843) (Hymenoptera: Cephidae) and its phylogenetic analyses

**DOI:** 10.1080/23802359.2022.2124828

**Published:** 2022-09-28

**Authors:** Mengmeng Liu, Zemin Sun, Özgül Doğan, Meicai Wei, Lin Liu

**Affiliations:** a College of Ecology, Lishui University, Lishui, China; b College of Life Sciences, Jiangxi Normal University, Nanchang, China; c Department of Molecular Biology and Genetics, Faculty of Science, Sivas Cumhuriyet University, Sivas, Turkey; d College of Forestry, Central South University of Forestry and Technology, Changsha, China

**Keywords:** Mitochondrial genome, phylogenetic analysis, Cephidae, *Neosyrista*, cryptic species

## Abstract

The complete mitochondrial genome of *Syrista parreyssii* (Spinola, 1843) was described. The circular genome is 18,666 bp with an A + T content of 82.60%. It contains 37 genes and a 1921 bp control region. The CR-*trnI* (+)-*trnQ* (–)-*trnM* (+) cluster is rearranged as *trnM* (+)-CR-*trnQ* (–)-*trnI* (+) cluster. Phylogenetic analysis demonstrates that European *Syrista* and Asian *Neosyrista* were not sister groups. *Neosyrista* is a valid genus and should be reestablished. Moreover, a preliminary study based on COI showed there are at least three valid *Syrista* species within the European and Mediterranean regions. Whether the known *Syrista parreyssii* (Spinola, 1843) is a complex or there are more cryptic species needs further study.

*Syrista* Konow (1896) now includes one European species, *Syrista parreyssii* (Spinola 1843), and four eastern Asian species. Benson (1935) ever erected *Neosyrista* Benson 1935 for the Japanese species, *Syrista similis* (Mocsáry 1904), which was the only Asian species of *Syrista* then. But Benson later merged *Neosyrista* with *Syrista* Konow (1946). Wei and Nie (1996), Wei (2007), and Wei and Smith (2010) studied the genus *Syrista* and added three additional species from eastern Asia. The most obvious morphological difference between the Asian species and European type species of the genus is the presence or absence of the anal cross vein in the fore wing, besides several other differences. The vein is absent in *S. parreyssii* and present in the four Asian species. *S. parreyssii* is one of the most important pests of *Rosa canina* and *R. damascena*. The larvae of *S. parreyssii* bore the shoots (Tozlu et al. 2017). In this study, we sequenced the mitochondrial genome of *S. parreyssii* and inferred the phylogeny of Cephidae to clarify the systematic position of *Syrista* and the relationship between *Syrista* and *Neosyrista*.

The specimen of *S. parreyssii* was deposited at the Asia Sawfly Museum, Nanchang (ASME) (Meicai Wei, weimc@126.com) under the voucher number CSCS-Hym-MC0234, which was collected in Sivas Cumhuriyet University Campus, Sivas, Turkey (39.705 N 37.026 E) on 3 June 2018, and identified by Meicai Wei. Whole genomic DNA was extracted from the specimen (CSCS-Hym-MC0234) by using the DNeasyR Blood & Tissue Kits (Qiagen, Valencia, CA). Genome sequencing was performed by using the high-throughput Illumina Hiseq 4000 platform, the genomic DNA sequences from 338,731,040 raw reads (SRR15850959) were assembled using MitoZ (Meng et al. 2019) and verified by Geneious Prime 2019.2.1 (https://www.geneious.com). With the invertebrate mitochondrial code, the assembled mitogenome was annotated using the MITOS web server (Bernt et al. 2013). In order to investigate the phylogenetic relationships of *S. parreyssii*, mitochondrial genome sequences of 17 species in the family of Cephidae were analyzed together. Multiple alignment of these sequences was performed using the MAFFT method in the TranslatorX server (Abascal et al. 2010). Partitioning schemes and models were estimated by using Phylosuit (Zhang et al. 2020). The maximum-likelihood (ML) tree was inferred with IQ-TREE (Nguyen et al. 2015) using GTR + I+G model, and the Bayesian inference (BI) tree was inferred with Mrbayes (Ronquist et al. 2012) under the GTR + I+G model.

The sequence yield by MitoZ was 18,619 bp in length and contained 13 protein-coding genes (PCGs), 22 tRNA genes, two rRNA genes, and an incomplete control region (CR). The obtained sequences were thoroughly examined by reassembly using *Stenocephus shenyang* (unpublished) and *Phylloecus fuscicosta* (unpublished) as reference sequences (coverage was 22,092 and 22,109, respectively). A 46 bp overlap was found in the incomplete CR. Using trnM, trnQ, and the 46 bp overlap as reference to reassemble and obtain the CR. After manual verification, the CR was 1921 bp in length. It was used as a reference to verify the reliability of the results, and a high-quality mapping with the flanking tRNAs was found.

The circular genome is 18,666 bp, including 37 genes and a complete CR. Compared with the putative ancestral gene arrangement of insects (Boore 1999), the CR-*trnI* (+)-*trnQ* (–)-*trnM* (+) cluster is rearranged as *trnM* (+)-CR-*trnQ* (–)-*trnI* (+), and in agreement with almost all Cephidae mitogenomes reported previously (e.g. Dowton et al. 2009; Korkmaz et al. 2016; Korkmaz et al. 2017). However, the gene order is different from *Syrista parreyssii* (KX907847). In the latter, *trnM* (+)-*trnA* (–)-*trnI* (+)-*trnQ* (–) cluster was detected, which is the only case reported in Cephidae. Besides, *trnP* is absent, and *trnT* is directly connected to the flanking PCGs (Korkmaz et al. 2018).

The A + T content of the whole mitochondrial genome is 82.60% (40.70% A, 11.20% C, 6.20% G, and 41.90% T), indicating significant A + T bias. Four start codons for PCGs are used, ATG (*atp6, cox2, cob, nad4, nad4l, nad5,* and *nad6*); ATA (*nad3*); ATT (*atp8, cox1, cox3,* and *nad1*) and ATC (*nad2*). All PCGs use TAA as a stop codon except for *nad5* (TAG). Part of *nad4* (from position 1308 to position 1353) of *S. parreyssii* (KX907847) has the difference in aligning when conducting the multiply alignment of 17 Cephidae, whereas, *S. parreyssii* (OK104785) reported here has no such problem.

Trees for BI and ML were the same in topology; Figure 1 shows the BI tree with nodal supports. Phylogenetic inference fully resolved *S. parreyssii* as a basal branch of Hartigiinae of Cephidae (Figure 1). While *Neosyrista incisa* (Wei & Nie 1996) was a sister group of *Janus megamaculatus* Liu & Wei, 2017 (Liu et al. 2017) and it was not a sister group of *S. parreyssii*. Besides, the monophyly of the genus *Janus* and the tribe Pachycephini, which was erected by Benson (1946) and composed of *Characopygus* and *Pachycephus*, were also problematic as shown in Figure 1. *Characopygus* was a sister group of (*Cephus*+*Trachelus*) and a member of Cephinae. While *Pachycephus* was a sister group of *Phylloecus* and a member of Hartigiinae. The phylogenetic relationship of the genera in the family Cephidae needs more sampling to be clarified.

**Figure 1.**
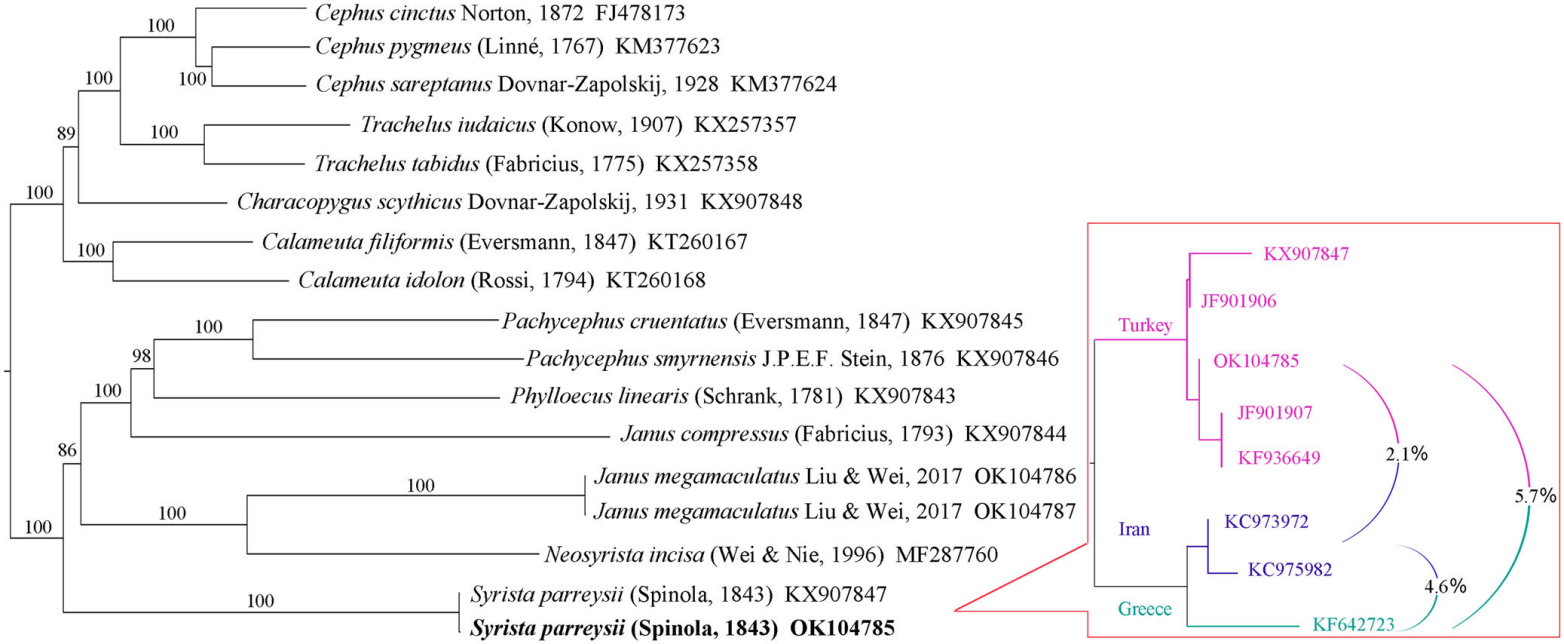
A Bayesian inference (BI) tree based on 17 Cephidae sequences from the 13 PCGs (left). Numbers on the branches correspond to Bayesian posterior probabilities. The accession number is given after each species. A maximum-likelihood (ML) tree based on eight *COI* sequences of *Syrista* species (right). P-distances between clades are provided.

Phylogenetic reconstruction using the barcoding sequence showed that the eight samples were divided into three branches corresponding to their collection places: Turkey, Iran, and Greece. The genetic distances between these three branches were 2.1%, 5.2%, and 4.3%, respectively, as in Figure 1. While no genetic distance was found within the clades. This result clearly shows that at least three species are bearing the name of *S. parreyssii*. However, this species is recorded in many localities (Wei and Smith 2010), spanning the Mediterranean Region, and reaching into the Caucasus. Therefore, it is possible that there are more species and provokes further questions.

The accumulation of the mitochondrial genome exposes inaccurate taxonomy problems (Yang et al. 2021), which will promote the progress of integrative taxonomy to a certain extent. Combining morphological and molecular evidence, more and more crypt species will be discovered. An increasing sample matrix makes it possible to construct genus-level phylogeny, thus providing conditions for solving the problem of complex species.

## Ethics statement

The collection of specimen conformed to the requirement of International ethics, which are unrestricted species. The collection was approved by the local authorities. The process and purpose of this experimental research were in line with the rules and regulations of our institute. There are no ethical issues and other conflicts of interest in this study.

## Author contributions

Conceptualization, M.W.; methodology, M.W.; validation, Ö.D.; formal analysis, Z.S., and M.L.; investigation, M.L.; resources, M.W.; data curation, M.L.; writing-original draft, M.L. and L.L.; writing-review and editing, M.L. and Ö.D.; visualization, L.L. and Z.S.; supervision, M.W.; project administration, M.W.; funding acquisition, M.L. and M.W. All authors have read and agreed to the published version of the manuscript.

## Data Availability

The genome sequence data that support the findings of this study are openly available in GenBank (https://www.ncbi.nlm.nih.gov) under the accession number OK104785 and gb file in Science Data Bank under the DOI:10.11922/sciencedb.01114. The associated BioProject, SRA, and BioSample numbers are PRJNA761906, SRR15850959, and SAMN21247251, respectively. All related files had been uploaded to figshare (https://figshare.com/account/home#/projects/123556).
